# Rapid research and implementation priority setting for wound care uncertainties

**DOI:** 10.1371/journal.pone.0188958

**Published:** 2017-12-05

**Authors:** Trish A. Gray, Jo C. Dumville, Janice Christie, Nicky A. Cullum

**Affiliations:** 1 Division of Nursing, Midwifery and Social Work, School of Health Sciences, University of Manchester, Manchester, United Kingdom; 2 Research and Innovation Division, Central Manchester University Hospitals NHS Foundation Trust, Manchester, United Kingdom; Geisinger Health System, UNITED STATES

## Abstract

**Introduction:**

People with complex wounds are more likely to be elderly, living with multimorbidity and wound related symptoms. A variety of products are available for managing complex wounds and a range of healthcare professionals are involved in wound care, yet there is a lack of good evidence to guide practice and services. These factors create uncertainty for those who deliver and those who manage wound care. Formal priority setting for research and implementation topics is needed to more accurately target the gaps in treatment and services. We solicited practitioner and manager uncertainties in wound care and held a priority setting workshop to facilitate a collaborative approach to prioritising wound care-related uncertainties.

**Methods:**

We recruited healthcare professionals who regularly cared for patients with complex wounds, were wound care specialists or managed wound care services. Participants submitted up to five wound care uncertainties in consultation with their colleagues, via an on-line survey and attended a priority setting workshop. Submitted uncertainties were collated, sorted and categorised according professional group. On the day of the workshop, participants were divided into four groups depending on their profession. Uncertainties submitted by their professional group were viewed, discussed and amended, prior to the first of three individual voting rounds. Participants cast up to ten votes for the uncertainties they judged as being high priority. Continuing in the professional groups, the top 10 uncertainties from each group were displayed, and the process was repeated. Groups were then brought together for a plenary session in which the final priorities were individually scored on a scale of 0–10 by participants. Priorities were ranked and results presented. Nominal group technique was used for generating the final uncertainties, voting and discussions.

**Results:**

Thirty-three participants attended the workshop comprising; 10 specialist nurses, 10 district nurses, seven podiatrists and six managers. Participants had been qualified for a mean of 20.7 years with a mean of 16.8 years of wound care experience. One hundred and thirty-nine uncertainties were submitted electronically and a further 20 were identified on the day of the workshop following lively, interactive group discussions. Twenty-five uncertainties from the total of 159 generated made it to the final prioritised list. These included six of the 20 new uncertainties. The uncertainties varied in focus, but could be broadly categorised into three themes: service delivery and organisation, patient centred care and treatment options. Specialist nurses were more likely to vote for service delivery and organisation topics, podiatrists for patient centred topics, district nurses for treatment options and operational leads for a broad range.

**Conclusions:**

This collaborative priority setting project is the first to engage front-line clinicians in prioritising research and implementation topics in wound care. We have shown that it is feasible to conduct topic prioritisation in a short time frame. This project has demonstrated that with careful planning and rigor, important questions that are raised in the course of clinicians’ daily decision making can be translated into meaningful research and implementation initiatives that could make a difference to service delivery and patient care.

## Introduction

In the past, health research agendas were driven largely by academic communities without direct consultation with those providing and receiving healthcare [1]. More recently there has been a growing emphasis on professional, patient and public involvement in the prioritisation of uncertainties [2]. We employed a collaborative approach to planning future research and implementation programmes by facilitating a wound care priority setting project involving key stakeholders. Knowing which uncertainties are important to patients and professionals matters as they may be different from those of researchers and funders [3]. A collaborative process for gathering uncertainties and setting research and implementation priorities is ethically desirable and can improve the quality and relevance of research and healthcare [4]. Fostering ownership of research findings can also promote meaningful translation of findings into clinical practice and service delivery.

People with complex wounds (wounds with superficial, partial or full thickness skin loss, healing by secondary intention with additional features such as exudate or infection) [5] e.g. leg ulcers, pressure ulcers and foot ulcers are more likely to be elderly [6] living with multimorbidity [7] and wound related symptoms such as pain [8], stress [9] and depression [10]. Cullum et al (2016) found that 73% of patients with wounds in the UK are cared for in community settings by a range of healthcare professionals (HCPs) and NHS services [11]. Dowsett et al (2014) found that approximately 70% of wound care is carried out in patients’ own homes [12]. Usual management of complex wounds involves the application of bandages, dressings and topical treatments. The range of wound care products is vast and much of the training provided for healthcare professionals on application, choice and management is provided by the pharmaceutical industry [13–15]. For profit organisations have also funded a high proportion of wound care research studies (41% from 2004–2011) [16], collectively resulting in a scarcity of clearly independent evidence. Additionally, the quality of the evidence for the effects of wound treatments is generally low [16]. The lack of good evidence, huge product choice, complex patient needs and fragmentation of care creates uncertainty regarding treatment plans. Involving those who deliver and those who manage wound care in exploring priorities may highlight more accurately the gaps in treatment and services. Moreover busy practitioners are frequently unaware of good research evidence that exists [17–19] and clinical uncertainties they identify may be important opportunities for research implementation if good evidence exists. We found a previous study that aimed to identify research and education priorities in wound management and tissue repair from the perspective of international experts i.e., people with a declared interest in wound healing (members of wound care organisations and delegates at wound care conferences including clinicians and academics) [20]. We could not identify any previous prioritisation exercise that involved front-line, non-specialist clinicians nor one that also addressed priorities for implementation.

The James Lind Alliance (JLA) supports collaborative partnerships between national charities or patient groups and professional organisations in identifying and prioritising treatment uncertainties associated with specific conditions and diseases [21]. To date there have been priority setting partnerships in a diverse range of conditions including pressure ulcers [22], epilepsy [23], eczema [3], urinary incontinence [24], type 1 diabetes [25] and CKD [26]. The majority of JLA projects have involved a multi-stage process of gathering uncertainties, collating and sorting of uncertainties to remove duplicates and those already answered by research, culminating in a prioritisation workshop. The process is very labour-intensive, lengthy and requires substantial funding. By contrast Tong et al [26] propose a more rapid approach to gathering and prioritising uncertainties within a 1-day workshop. This latter approach does not remove those uncertainties which are already “answered;” this was attractive to us as we also wanted to identify uncertainties raised by participants which have been substantially reduced, or eliminated by research, for future research implementation work. This paper describes a research and implementation prioritisation process that was a hybrid of the JLA and the 1-day workshop approach [1, 26] involving healthcare professionals (HCPs) and managers who manage complex wounds and wound care services in community settings. This project is part of a wider programme of wound care work developed by the Collaboration for Leadership in Applied Health Research and Care Greater Manchester (CLAHRC GM) and funded by the National Institute for Health Research (NIHR) with matched funding from four partner community NHS Foundation Trusts: Central Manchester University Hospitals NHS Foundation Trust (CMFT), Pennine Care NHS Foundation Trust (Pennine Care), Salford Royal NHS Foundation Trust (SRFT), University Hospital of South Manchester NHS Foundation Trust (UHSM). Our four Northern England, partner NHS Trusts provide care for a population of 1.5 million across rural and urban locations within Greater Manchester which itself has a population of 2.7 million.

## Materials and methods

**Fig 1:**
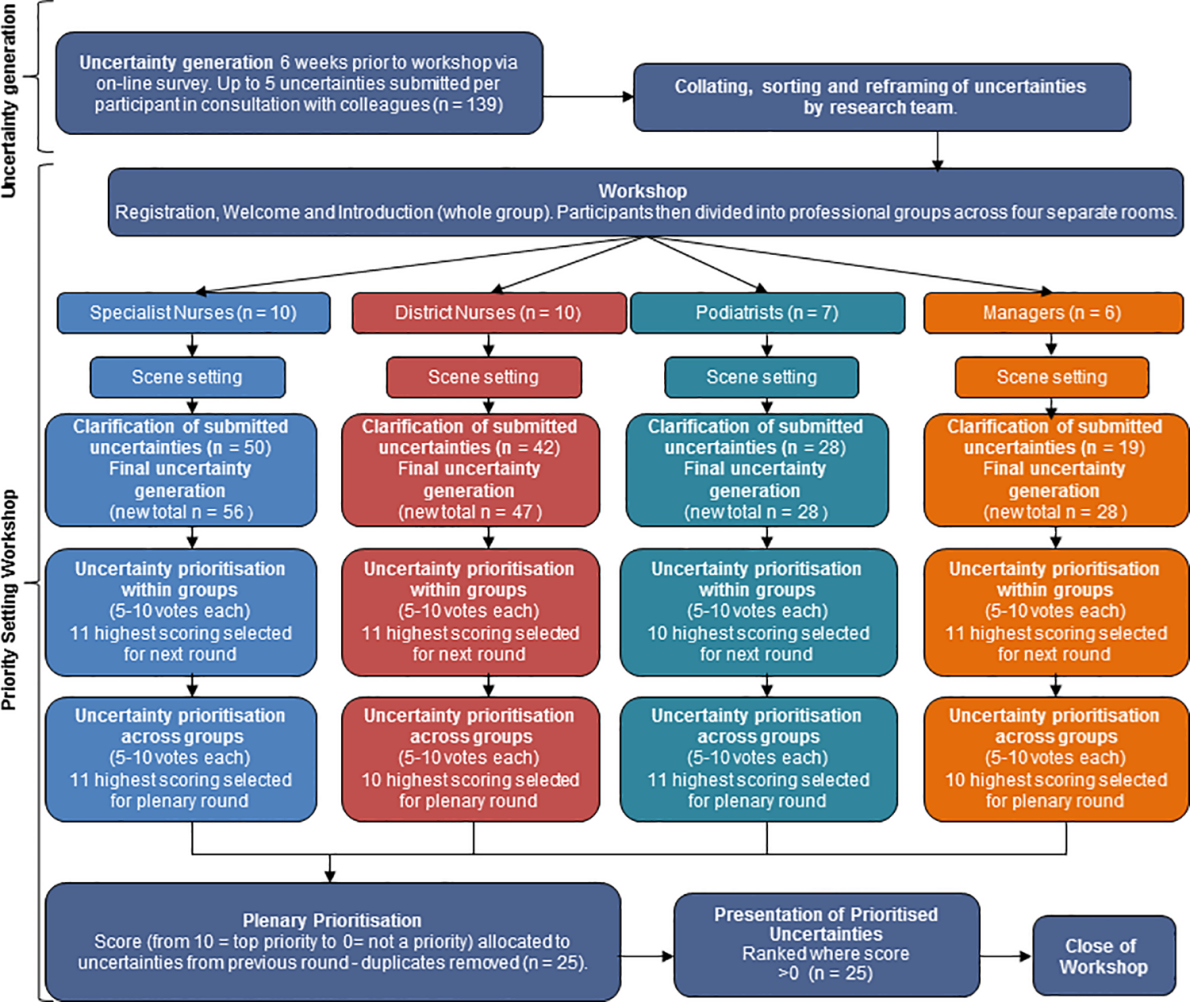
Methods flow chart.

We solicited wound care uncertainties from wound care practitioners and managers and subsequently considered and prioritised these together in a one-day priority setting workshop. The flow diagram in Fig 1 presents the steps as described below.

### Participant recruitment

Purposive sampling was used to ensure that participants had relevant clinical and management experience and expertise. NHS HCPs were eligible to participate if they regularly cared for patients with complex wounds, were wound care specialists or managed wound care services. Interested potential participants were identified through contacts developed at the inception of the NIHR CLAHRC Greater Manchester wound care programme and were approached via email, telephone or via a face-to-face meeting. We aimed to recruit between 6 and 12 participants from each of the following four professional groups: district nurses, podiatrists, wound care specialists and service managers; based on sample recommendations from existing literature [27]. More district nurses were invited than other professional groups to take account of the size of their workforce and contribution to wound care. An additional 50% of participants over and above the number of six per group suggested by Carney et al (1996) were invited to allow for withdrawals [28].

Ethics approval from a local Research Ethics Committee was not required for this work as we recruited HCPs to capture their thoughts and views in the context of their day-to-day professional roles. Ethics approval was sought and granted from the University of Manchester Research Ethics Committee and Research and Development Approval obtained from all partner Trusts. Written informed consent was obtained from all participants. A Participant Information Sheet was emailed to potential participants to provide information about the workshop with detailed instructions for generating and submitting uncertainties.

### Data collection

Participants were asked to identify up to five uncertainties about wound care in consultation with their colleagues and submit these six weeks prior to the workshop via an on-line survey. Supporting material provided guidance on formulating uncertainties using the PICO framework [29, 30]. Participants were encouraged to submit in alternative formats if they felt that PICO did not fit their uncertainty. A summary of the guidance is provided in Box 1 with examples of prioritised uncertainties from other authors [3, 23, 26, 31, 32]. Unlike the JLA approach, we did not require the uncertainties to be about the effectiveness of interventions and welcomed uncertainties on any wound care issue.

**Box 1. Uncertainty gathering guidance**

**What are uncertainties?**

By uncertainties, we mean aspects of wound care where you are not sure of the best way to proceed and where research evidence may help to reduce the uncertainty. These uncertainties or “unanswered questions” may be about patient problems, best treatments or how wound care services are delivered. Here are a few examples:

- Does wound healing differ in patients with different diseases?
- Does the frequency of dressing changes affect wound healing?
- Which type of healthcare professional is most suited for wound assessment?
- What is the optimum skill mix for managing community wound clinics?

**Defining uncertainty questions**

Once you have an idea of the type of uncertainties you have, try to define them to make them more specific. It may help to use a PICO format. PICO is a taxonomy used in evidence based healthcare to help formulate research questions the acronym stands for:

- **P**atient, population or process of interest,
- **I**ntervention, management, test or best practice to be assessed,
- **C**omparator, another intervention or test or usual care
- **O**utcome or effect of interest i.e. to improve survival or quality of life, to reduce complications or pain.

**A PICO question may look like this**:

- Does weekly monitoring of blood pressure **(I)** in patients with diabetes **(P)** maintain target BP **(O)** more effectively than two weekly monitoring **(C)**?

**Questions that do not fit the PICO format**

You may be able to fit your question to two or three of the PICO criteria. You may think of a **P**atient group, an **I**ntervention and/or an **O**utcome but the **C**omparator component may not fit the question, some examples are:

- Do patients’ beliefs about adherence to therapy limit treatment options?
- How can we use diabetic patients’ experiences to improve the management of hypoglycemia?

We have offered PICO as a building block to help you to develop uncertainty questions by breaking your ideas into individual elements then combining them to form a potential research question but we know that not all research questions fit this structure, so please feel free to submit all questions. We certainly want to avoid stifling your thoughts. We would like to capture all uncertainties regardless of structure.

**Other examples of uncertainties from other areas of healthcare**

Here are a few more examples of questions. We have purposely not provided examples from wound care as we do not wish to influence your thoughts:

- Do shared electronic patient records improve communication across integrated health and social care teams?
- Does active implementation of clinical practice guidelines in general practice improve kidney health in patients with early CKD?
- Does implementing a personalised care plan increase quality of life for patients with breast cancer compared to standard care?
- Do anxious patients with eczema have shorter symptom free periods than non-anxious patients?
- What factors influence the development of refractive error (myopia, astigmatism, presbyopia and long-sightedness)?
- Does early mobolisation and physiotherapy after shoulder surgery improve patient outcomes compared to standard immobilisation and physiotherapy?
- Can better education about epilepsy improve quality of life for people with epilepsy by reducing stigma?

All submitted uncertainties were collated, sorted and categorised according professional group and entered onto an Excel spread sheet (Microsoft Office 2007). Duplicate uncertainties from the same professional group were removed, but uncertainties duplicated across different groups were retained. Where appropriate and feasible, we re-framed uncertainties into the PICO format if they had not been submitted using PICO. At least two researchers checked each uncertainty against the original submission. Discrepancies were resolved by discussion.

On the day of the workshop, all participants were brought together for a brief introduction before being divided into four groups depending on their profession or role, viz: specialist nurses, district nurses, podiatrists and managers. Each group was shown to a separate room by their lead and co-facilitator. All lead facilitators were researchers with group facilitation experience. A third team member in each group provided administrative support. Facilitators and the support team worked from a standard operating procedure (SOP) developed in advance and shared during a preparatory training session involving a mock-up of the workshop. Group-specific submitted and sorted uncertainties were printed out individually and displayed on a wall within that group’s room. To allow participants time to study these independently, uncertainties were also printed on A4 sheets and handed to participants.

#### Uncertainty prioritisation within groups

Participants viewed the uncertainties submitted to ensure that they recognised their own and understood the others. Participants were given the opportunity to amend the wording of uncertainties to clarify their meaning and submit one more uncertainty each if they felt there was an important omission; time was provided for discussion. New or amended uncertainties were printed out and displayed. Nominal group technique (NGT) was used for generating the final uncertainties, voting and discussions. NGT is a structured face-to-face group interaction which empowers participants by providing the opportunity to have their voices heard and opinions considered by other members [33]. It usually consists of four stages: silent generation, round robin, clarification and voting; giving participants an equal chance to contribute without pressure to conform to individual viewpoints [34].

Participants were then given 10 coloured stickers each and were asked to place between five and 10 stickers on the uncertainties they deemed to be the most important, using only one sticker per uncertainty. Once selections were complete, the votes were counted and the top 10 scoring uncertainties were taken through to the next round. Ties were resolved by giving participants one further sticker to allocate to any uncertainty. Any further tied uncertainties were taken through to the next round. Time was allocated for reflection and a review of choices.

#### Uncertainty prioritisation across groups

Continuing in the professional or role-based groups, copies of the top 10 uncertainties from each group were printed on A4 sheets and distributed to each participant and also displayed on posters in every room. Duplicates were removed following discussion and agreement across all groups (a runner coordinated this consensus process). Again, time was provided for clarification and discussion prior to commencing round two, ‘Uncertainty prioritisation across groups’. The voting process (again using stickers) was repeated after which participants reflected on the results and reviewed their choices.

The voting process for the first two rounds was conducted in real time. The support team used laptops to enter their group’s votes into an Excel spread sheet (Microsoft Office 2007) located on a shared drive. The spread sheet was accessed and collated by a data analyst (housed in a fifth room), prior to printing the refined uncertainty lists in preparation for the next round. Support team members acted as runners between the analyst and facilitators; collecting and distributing newly printed uncertainty lists.

#### Plenary session and ranking of uncertainties

The four groups were brought together for a plenary session in which final priorities were presented, discussed and agreed. As before, participants individually reviewed the uncertainties prior to a group discussion to resolve any outstanding issues. The collated priorities from the previous round were printed onto score sheets and participants were asked to individually score each uncertainty on a scale of 0–10 (0 = not a priority and 10 = a top priority). Once scoring was complete, priorities were ranked by the analyst and checked by a member of the research team prior to presenting the participants with the results. Uncertainties with scores >0 were presented.

### Data analysis

The prioritised lists were entered into Excel (Microsoft Office 2007) for real time recording, calculation of votes and plenary scores. Following the workshop, data were transferred to SPSS (IBM; version 22) for descriptive analysis. As data were not normally distributed, medians and IQRs were calculated for each uncertainty in the top 25. Uncertainties were stratified by participant group (profession or role) and a Mann-Whitney U test was conducted to test for differences between groups.

## Results

### Participant characteristics

Thirty-one of the 76 healthcare professionals we contacted submitted uncertainties. Thirty-three attended the workshop (43% attendance). Reasons for non-attendance included work commitments and illness. Across the four community NHS Trusts, attendance was fairly evenly spread. Ten specialist nurses attended (mostly tissue viability nurses); 10 district nurses, seven podiatrists and six managers covering podiatry, pharmacy and community nursing. Only three participants (9%) were male. Participants were very experienced healthcare professionals (mean years since qualification 20.7; SD 10.2) with extensive wound care experience (mean 16.8 years, SD 10.3). Thirteen (39%) had a wound care accreditation and 23 (70%) had attended a wound care course within the last 12 months (Table 1).

**Table 1:** Participant demographic details.

Participant Demographics	(n = 33)
***Characteristics***	***N (%)***
***Gender***	
Male	3 (9)
Female	30 (91)
***Role group***	
Specialist Nurse with tissue viability expertise	10 (30.3)
District nurse	10 (30.3)
Podiatrist	7 (21.2)
Manager	6 (18.2)
**Highest Academic Qualification**	
PhD	1 (3.0)
MSc	4 (12.4)
PGDip	12 (36.4)
PGCert	5 (15.2)
BSc	7 (21.2)
Vocational Qualification	4 (12.1)
***Wound care accreditation***	
Yes	13 (39.4)
No	20 (60.6)
***Attended a wound care course in the last 12 months***	
Yes	23 (69.7)
No	10 (30.3)
***Research Experience***	
Yes	20 (60.6)
No	13 (39.4)
***Years of experience***	**Mean (SD) Range**
Since professional qualification	20.7 ***(***10.2) 4 to 40
Managing wounds	*16*.*7* (10.3) 0 to 38

### Priority setting

One hundred and thirty-nine uncertainties were submitted electronically pre-workshop and a further 20 were identified on the day of the workshop. Twenty-five uncertainties from the total of 159 generated before and during the morning session made it to the final prioritised list (Table 2). These included six of the 20 new uncertainties identified on the day. A fairly even spread of uncertainties from each professional group reached the final 25; five from district nurses, six from managers, eight from podiatrists and six from the specialist nurses.

**Table 2:** Top 25 wound care uncertainties.

Uncertainty	Overall *Median score (IQR)*	Specialist nurses *Median score (IQR)*	District nurses *Median score (IQR)*	Podiatrists *Median score (IQR)*	Managers *Median score (IQR)*
**D**oes patient involvement in their dressing changes improve outcomes or increase negative outcomes?	9.0 (7.00–9.50)	9.0 (6.50–10.00)	7.5 (5.75–9.00)	8.0 (7.50–9.00)	9.0 (7.00–10.00)
**W**hat is the most reliable and valid method of grading pressure ulcers?	9.0 (6.00–10.00)	10.0 (8.50–10.00)	8.5 (5.00–10.00)	7.0 (4.00–8.50)	7.0 (4.50–9.00)
**W**ould standardising wound assessments and tools across NHS settings improve staff productivity and patient outcomes?	8.0 (7.00–10.00)	9.0 (7.50–10.00)	9.0 (7.50–10.00)	7.0 (6.50–8.50)	8.0 (7.50–9.50)
**H**ow does nursing and/or professional skill mix influence wound outcomes in community settings? What training is required to best manage patients with complex wounds?	8.0 (7.00–9.00)	8.0 (6.50–9.50)	8.5 (7.50–9.25)	8.0 (7.00–9.00)	8.0 (4.50–8.50)
**D**o integrated team-based interventions aimed at better communication and collaborative working, improve patient outcomes?	8.0 (6.00–9.00)	8.0 (6.00–9.50)	7.5 (5.25–9.00)	9.0 (8.50–10.00)	7.0 (6.00–9.00)
**D**oes continuing professional development in wound care improve the quality of care and patient outcomes compared with no annual update?	8.0 (6.00–9.00)	8.0 (5.50–9.50)	8.5 (6.75–9.25)	9.0 (7.50–9.00)	6.0 (4.00–7.50)
**W**hat effects do electronic patient records have on patient and service outcomes across a wound care service compared to paper records?	8.0 (5.00–9.50)	7.0 (5.50–9.50)	8.5 (7.25–9.00)	7.0 (3.50–10.00)	5.0 (2.50–10.00)
**W**hich treatments are most effective for over granulation?	8.0 (5.00–9.00)	8.0 (5.50–9.50)	9.0 (7.25–9.25)	3.0 (1.00–5.00)	7.0 (5.50–9.00)
**W**hat is the most clinical and cost-effective criteria for referring to specialist services (e.g. tissue viability/podiatry) to ensure appropriate use of resources and referral time?	8.0 (5.00–9.00)	9.0 (8.50–9.50)	7.5 (6.75–8.50)	5.0 (2.50–6.00)	6.0 (4.00–9.00)
**H**ow do we differentiate between diabetic foot wounds and pressure ulcers? Does this influence management and outcomes?	8.0 (4.00–10.00)	8.0 (2.00–9.50)	6.5 (3.25–9.25)	10.0 (9.00–10.00)	8.0 (3.50–9.00)
**D**o patients with venous leg ulceration heal quicker when treated in a dedicated leg ulcer clinic compared with general community clinics?	8.0 (4.00–9.00)	8.0 (5.00–9.50)	8.5 (6.25–10.00)	0.0 (0.00–8.00)	9.0 (4.50–9.50)
**W**hat are the effects of different cleansing agents on infection and healing of wounds in community settings?	7.0 (6.00–9.00)	7.0 (6.00–7.50)	9.0 (6.25–10.00)	6.0 (4.50–7.00)	8.0 (7.00–9.00)
**W**hat are the clinical and cost effective methods for managing an excess of wound exudate?	7.0 (4.00–9.50)	6.0 (2.00–9.50)	9.0 (6.75–10.00)	4.0 (3.50–6.50)	9.0 (5.00–10.00)
**D**oes sharp debridement speed up wound healing in chronic wounds compared with dressings (HCL, hydrogels etc)?	7.0 (5.00–8.00)	7.0 (3.50–8.00)	7.5 (6.75–9.25)	8.0 (5.50–9.00)	7.0 (4.50–8.50)
**H**ow should we identify where biofilm is impeding wound healing? How should we manage it? How aware of biofilm are people involved in the management of wounds? What is the best way to manage a biofilm?	7.0 (5.00–8.00)	5.0 (1.50–6.50)	8.0 (6.00–10.00)	8.0 (7.50–10.00)	7.0 (6.00–7.50)
**H**ow do we promote adherence to interventions and health behaviours in people at high risk of foot problems?	7.0 (5.00–8.00)	7.0 (1.50–7.00)	8.0 (6.50–8.25)	9.0 (7.00–9.50)	6.0 (4.00–7.50)
**D**o anti-microbial containing wound dressings heal infected wounds more quickly than oral antimicrobials?	7.0 (4.50–9.00)	5.0 (4.50–8.50)	8.0 (5.25–10.00)	3.0 (1.00–8.00)	8.0 (7.50–9.50)
**D**oes a prescribed two week treatment plan, using the same type of dressing, affect healing outcomes versus ad-hoc dressing selection?	7.0 (4.50–9.00)	9.0 (6.00–10.00)	8.5 (6.25–9.00)	3.0 (2.50–5.00)	6.0 (4.50–6.50)
**W**hat is the best way of cleaning venous leg ulcers in terms of promoting healing and preventing infection?	7.0 (2.50–7.00)	4.0 (2.50–7.50)	9.5 (7.25–10.00)	2.0 (1.00–5.00)	8.0 (7.00–9.00)
**D**o psychological interventions (i.e., aimed at changing health beliefs and behaviours) improve the healing/reduce the incidence of ulcers on the feet of people with diabetes?	6.0 (5.00–8.00)	5.0 (3.50–6.50)	6.0 (4.75–7.25)	8.0 (8.00–10.00)	5.0 (5.00–6.50)
**H**ow can accurate detection of clinical infection be facilitated across different skill mixes?	6.0 (4.50–9.00)	6.0 (3.00–7.50)	8.0 (4.25–9.25)	8.0 (6.50–9.50)	5.0 (3.00–8.00)
**W**hat should be used for infected wounds when the bacteria are resistant to antibiotics?	6.0 (4.50–8.50)	5.0 (3.00–5.50)	9.0 (7.50–10.00)	5.0 (1.00–7.00)	8.0 (6.50–9.50)
**D**oes off-loading for people with foot wounds Improve wound healing compared with usual (or increased) activity?	5.0 (3.50–7.00)	4.0 (2.50–5.00)	6.0 (4.25–7.25)	10.0 (8.50–10.00)	5.0 (2.50–6.00)
**W**hat impact do walk in centres have on patients outcomes versus treatment room clinics?	5.0 (1.00–8.00)	7.0 (3.00–8.00)	7.5 (4.00–10.00)	1.0 (0.00–4.50)	3.0 (0.50–8.00)
**D**oes stopping packing a sinus wound when it has healed to 1cm depth and then treating with medical honey speed wound healing compared with usual care?	5.0 (1.50–8.00)	2.0 (1.00–7.00)	7.5 (6.50–9.25)	2.0 (0.00–3.50)	7.0 (3.00–9.00)

Scores were allocated on a scale of 0–10 where 0 = not a priority and 10 = a top priority.

The uncertainties varied in focus, but could be broadly categorised into three themes: service delivery and organisation, patient centred care and treatment options. In the second round of voting (across groups), specialist nurses were more likely to vote for service delivery and organisation topics, podiatrists for patient centred topics (e.g., self-care), district nurses for treatment options and managers for a broad range. There was good agreement on priorities within the specialist nurse, district nurse and podiatrist groups. The managers group had heterogeneous membership and there was less agreement within the group and a greater range of lower scores.

In the final plenary session this pattern continued. District nurses gave the highest score to the uncertainty: “What is the best way of cleaning venous leg ulcers in terms of promoting healing and preventing infection?” (median score 9.5; IQR 7.25–10.00), whilst specialist nurses gave the highest score for a reliable and valid method for grading pressure ulcers (median score 10.0; IQR 8.50–10.00). Managers prioritised patient involvement in dressing changes (median score 9.0; IQR 7.00–10.00), management of wound exudate (median score 9.0; IQR 5.00–10.00) and the effect on healing of treating leg ulcers in a dedicated clinic rather than a general treatment room clinic (median score 9.0; IQR 4.50–9.50). An uncertainty related to off-loading versus usual activity for people with foot wounds was scored very highly by podiatrists (median score of 10.0, IQR 8.50–10.00) but significantly lower by other groups. Specialist nurses gave the lowest score for this uncertainty (median score 4.0, IQR 2.50–5.00, Z = -3.48, p < 0.001). Overall, the most highly prioritised uncertainties were:

- Does patient involvement in their dressing changes improve outcomes or increase negative outcomes? (median score 9.0; IQR 7.00–9.50)
- What is the most reliable and valid method of grading pressure ulcers? (median score 9.0; IQR 6.00–10.00)
- Would standardising wound assessments and tools across NHS settings improve staff productivity and patient outcomes? (median score 8.0; IQR 7.00–10.00)
- How does nursing and/or professional skill mix influence wound outcomes in community settings? What training is required to best manage patients with complex wounds? (median score 8.0; IQR 7.00–9.00)
- Do integrated team-based interventions aimed at better communication and collaborative working improve patient outcomes? (median score 8.0; IQR 6.00–9.00)
- Does continuing professional development in wound care improve the quality of care and patient outcomes compared with no annual update? (median score 8.0; IQR 6.00–9.00)
- What effects do electronic patient records have on patient and service outcomes across a wound care service compared to paper records? (median score 8.0; IQR 5.00–9.50)
- Which treatments are most effective for over granulation? (median score 8.0; IQR 5.00–9.00)
- What are the most clinically and cost-effective criteria for referring patients with complex wounds to specialist services (e.g. tissue viability/podiatry) to ensure appropriate use of resources and referral time? (median score 8.0; IQR 5.00–9.00)
- How do we differentiate between diabetic foot wounds and pressure ulcers? Does differentiation influence management and outcomes? (median score 8.0; IQR 4.00–10.00)
- Do patients with venous leg ulceration heal quicker when treated in a dedicated leg ulcer clinic compared with general community clinics? (median score 8.0; IQR 4.00–9.00)

At the end of the workshop participants were asked to complete an evaluation of the day. Thirty two participants completed the evaluation questionnaire. Responses were scored using a five-point Likert scale. Over half (59%, n = 19) rated the day as ‘Very good’ whilst the remaining 41% (n = 13) rated the day as ‘Good’. Participants were either very satisfied (72%, n = 23) or somewhat satisfied (28%, n = 9) that everyone had an equal chance of influencing the overall priorities. Ninety four percent (n = 30) reported that the workshop had increased their interest in wound care research. One participant valued the opportunity of being involved in a *“proactive bottom-up approach to supporting us (clinical professionals) in wound care”*, whilst another felt that working in small groups “*brought out the best in people”*, in terms of feeling at ease whilst participating during discussions.

## Discussion

It is essential that we spend scarce research resources on questions of importance to health and health services, to avoid research waste [35–38]. Funders should prioritise research that meets the needs of patients and healthcare professionals who deliver care. There is growing awareness that collaboration and consensus is required to better align research to the priorities of patients and clinicians [1, 2, 39].

This is only the second study we have identified which has engaged front-line clinicians in research prioritisation in wound care (the other was specifically related to pressure ulcers) [22] and the first with a goal of also identifying implementation topics. According to our knowledge, few studies have evaluated the sustainability of research implementation; available evidence suggests that sustainability relies upon continuous collaboration and joint ownership, addressing the needs of the target community, a balanced package of incentives and adequate funding [40–43]. Our approach is to develop enduring partnerships with the community services we serve. This will involve ongoing support, listening to feedback and equality in decision making to ensure that we can make a difference to wound care services, practice and patient care and that change remains relevant and sustainable.

We have shown that it is feasible to conduct topic prioritisation within a one-day collaborative workshop, providing this is extremely well-planned and based on some preparatory work by participants. We strived to deliver a comprehensive process, ensure inclusiveness, consider values and context and foster sustainable relationships with stakeholders beyond implementation of priorities; all key components of a successful priority setting process [38, 44]. Although our prioritisation was organised to unearth the priorities of HCPs in Greater Manchester (which comprises a population of 2.7 Million) we feel that our methods are transparent and replicable and many of the priorities raised (particularly those related to improving health professionals’ skills, improving patient access and patient self-management) are common across health fields [25, 26, 45–47] showing that our study has international relevance.

### Comparisons between the priorities of different professional groups and roles

There was good consensus between members for three of the groups; specialist nurses, district nurses and podiatrists regarding the highest priority uncertainties. During discussion, specialist nurses expressed a strong view of the need to improve nurses’ competency regarding wound assessment, pressure ulcer grading and timely referral for specialist support and these issues were also reflected in three of their top priorities. As patient care becomes increasingly complex due to the advancing age of patient populations, our need for healthcare professionals who are highly educated, skilled, critical thinkers is recognised not only in wound care but across healthcare [20, 48–50].

Podiatrists submitted the fewest uncertainties and added no new ones but had in-depth discussions about those submitted to ensure that the wording was a true reflection of their uncertainties; they were concerned that many were orientated towards nursing, and chose to generalise these, for example, they reworded nursing skill mix to professional skill mix and tissue viability to clinical specialist. Even though podiatrists submitted the fewest, the uncertainties they generated were influential, however, as eight of their uncertainties were voted into the top 25 (the largest number from a group).

It was inspiring to find that whilst there were no patients or carers present, a number of patient centred uncertainties reached the final 25 with one receiving the highest median score: “Does patient involvement in their dressing changes improve outcomes or increase negative outcomes?” It is particularly reassuring to find that similar patient centred priorities (self-management, access to services and multidisciplinary approaches to care) feature highly in other priority setting studies that did involve patients and carers [3, 25, 26, 51]. An important area not featured, however, was pain. Of the 159 uncertainties submitted only one was related to pain and this did not reach the final round. Wound related pain is well recognised as a debilitating symptom in people with chronic wounds [52], it was ranked as a top priority in an international eDelphi study of wound care experts (members of wound care societies and/or delegates at wound care conferences) conducted by Cowman et al [20] to prioritise wound care research topics and professional learning needs. Only one uncertainty relating to psychological factors reached the final top 25 although a number were submitted, again there is wide recognition that psychological factors including depression, stress and coping are associated with wound healing. [9, 10]. It would be interesting to see to which extent patient centred outcomes such as pain, depression and stress are highlighted by patients and carers. We had planned to repeat the process with patients and carers as participants, however, early indications suggest that this may prove difficult to do (as explained in more detail below). We will endeavour to reach out to this patient group by capturing their priorities using the most appropriate methodology, bearing in mind the barriers we face.

### Comparison with other priority setting projects

The James Lind Alliance (JLA) approach to priority setting was used as a framework for the design of this project; however, we have modified the process to achieve our aims. We did not include patients and carers unlike JLA partnerships [4, 24, 53] as the local nature of our exercise meant the HCPs involved were likely to deliver care to the patient participants. Furthermore because people with complex wounds are frequently old and frail it is very difficult to engage them in group activities of this nature. Our preliminary work with patients and carers in the NIHR CLAHRC Greater Manchester wounds programme has highlighted unwillingness amongst people with complex wounds to attend meetings or group activities such as this. Our previous JLA priority setting partnership in pressure ulcers found that those people able to engage in active research priority setting were younger people living with disabilities, carers and health professionals rather than older people with complex wounds [11]. Our next step will be to undertake some more in-depth interviews with people with complex wounds to better understand their perspectives on research, research priorities and engagement in research.

JLA priority setting projects are focused on intervention-related uncertainties, and therefore, use the PICO format. However, we wanted to capture any type of uncertainty felt important by participants. This broader approach gave the research team extra work reformulating uncertainties to ensure that they were comprehensible, whilst retaining the meaning, but it was worth it as we received far more uncertainties than we originally expected. This also led to deeper discussions at the start of the day ensuring that our rewording captured what participants had meant and potentially generated a number of new uncertainties as participants reflected on and explored missing concerns.

We did not restrict uncertainties reaching prioritisation to genuine uncertainties i.e. those not already answered by research. We wanted to see, as Tong et al did, [26] whether participants voted for uncertainties that were already answered by research as we felt this would highlight implementation priorities. One of the aims of our the wound research programme is to identify where current practice does not reflect the existing research evidence and where there is potential to make a difference to health services and healthcare, thereby, openly encouraging implementation of new, innovative wound care interventions as well as research opportunities. Since the prioritisation workshop we have reviewed the research literature to clarify which are true uncertainties where systematic reviews or primary research is needed, and which are uncertainties which have been wholly or partially eradicated by research. An analysis of wounds research as it relates to practitioner priorities will be the subject of a separate paper.

Tong et al [26] succeeded in completing their entire prioritisation process in one day; we chose to take a little longer (asking participants to submit uncertainties in advance of the one-day workshop) so that we could enable input from services and individual clinicians who could not attend on the day. We asked participants to involve their colleagues in generating uncertainties to provide a team rather than an individual perspective, this we felt would also assist the services involved to maintain ownership of the project. We found a number of HCPs who could not attend were very interested in the project and independently submitted uncertainties, asking to be included in the circulation of findings. Submitting uncertainties prior to the workshop also gave participants thinking time that aided early engagement with discussions on the day. To guarantee that all professional groups had equal opportunity to define and decide priorities, we ensured that panels were uni-professional during the early voting rounds. This was further supported by housing the uni-professional groups in separate rooms. We learned, however, that good communication between groups was essential during ‘uncertainty prioritisation across groups’ as participants sought to amalgamate and amend uncertainties. We facilitated this iterative process by using the support team runners. In addition, we found that we needed more time than planned to calculate the final ranking scores before presenting these to the participants; one solution would have been to disseminate the results post-workshop, but as we wanted to conclude with a presentation of the final scores, we overcame this with an extended tea break.

In common with others [54–56] we used purposive sampling for recruitment as we wanted to maximise the range of healthcare professionals with wound care experience. We endeavoured to ensure that we had representation from the key community wound care decision makers by building good relationships with HCPs and their managers. This was done through pre-workshop meetings to cultivate a culture of openness, transparency and partnership. We provided detailed information about the importance of their contribution, how to define an uncertainty and offered feedback to participants and their managers throughout the process. Having a good knowledge of our target population and a good understanding of the current NHS pressures we knew that there would be a high non-respondent and withdrawal rate. We still had a least six participants per group as suggested by Carney et al (1996) to help avoid participants feeling vulnerable during group discussions [28]. In addition, as we had planned for drop out, no initial discussion group size exceeded 12 as advised by Harvey and Holmes (2012) [27].

In general terms our top priorities match other healthcare priority setting projects in terms of choosing effective treatments to improve clinical outcomes, self-management, multidisciplinary working, and staff and patient education [3, 26, 32, 47, 57, 58]. Although based on a Greater Manchester population, we believe our methodological approach has international relevance and it is likely the priorities we derived will be relevant in much of the developed world.

## Conclusions

This collaborative priority setting project has demonstrated that with careful planning and rigor, important uncertainties experienced by wound care practitioners and managers can be translated into meaningful research and implementation initiatives. If these research questions were answered and existing evidence was implemented we believe there would be beneficial outcomes for patients with complex wounds and likely important savings for health care providers. Our next steps involve taking forward research priorities into up to date systematic reviews (where needed) and/or new primary research to reduce the uncertainty. Any reviews will be conducted as part of the Cochrane Collaboration [59] to ensure international access to the findings. Where we know there is already good quality, relevant research evidence, we plan to embark on implementation projects, in collaboration with practitioners and managers. We continue to work closely with our NHS partners in our endeavour to undertake high quality wound care research and implementation projects to reflect the priorities of healthcare professionals and the patients they care for.
